# Tigecycline Dosing Strategies in Critically Ill Liver-Impaired Patients

**DOI:** 10.3390/antibiotics11040479

**Published:** 2022-04-03

**Authors:** Lisa F. Amann, Rawan Alraish, Astrid Broeker, Magnus Kaffarnik, Sebastian G. Wicha

**Affiliations:** 1 Department of Clinical Pharmacy, Institute of Pharmacy, University of Hamburg, 20146 Hamburg, Germany; lisa.amann@uni-hamburg.de (L.F.A.); broeker.astrid@gmail.com (A.B.); 2 Department of Surgery, Charité-Universitätsmedizin Berlin, 13353 Berlin, Germany; rawan.alraish@charite.de (R.A.); mkaffarnik@posteo.de (M.K.)

**Keywords:** population pharmacokinetics, Child–Pugh score, dose adjustment

## Abstract

This study investigated tigecycline exposure in critically ill patients from a population pharmacokinetic perspective to support rational dosing in intensive care unit (ICU) patients with acute and chronic liver impairment. A clinical dataset of 39 patients served as the basis for the development of a population pharmacokinetic model. The typical tigecycline clearance was strongly reduced (8.6 L/h) as compared to other populations. Different models were developed based on liver and kidney function-related covariates. Monte Carlo simulations were used to guide dose adjustments with the most predictive covariates: Child–Pugh score, total bilirubin, and MELD score. The best performing covariate, guiding a dose reduction to 25 mg q12h, was Child–Pugh score C, whereas patients with Child–Pugh score A/B received the standard dose of 50 mg q12h. Of note, the obtained 24 h steady-state area under the concentration vs. time curve (AUC_ss_) range using this dosing strategy was predicted to be equivalent to high-dose tigecycline exposure (100 mg q12h) in non-ICU patients. In addition, 26/39 study participants died, and therapy failure was most correlated with chronic liver disease and renal failure, but no correlation between drug exposure and survival was observed. However, tigecycline in special patient populations needs further investigations to enhance clinical outcome.

## 1. Introduction

Tigecycline, belonging to the class of glycylcyclines, is a last-resort antibiotic and currently approved for complicated skin and skin structure infections (cSSI), complicated intra-abdominal infections (cIAI), and community-acquired pneumonia. Its broad spectrum includes Gram-negative and Gram-positive strains, as well as multidrug-resistant pathogens [1]. Tigecycline is considered a bacteriostatic drug that inhibits the bacterial protein translation by binding to the 30S ribosomal subunit [2]. The 24 h steady-state area under the drug concentration vs. time curve (AUC_ss_) to minimum inhibitory concentration (MIC) ratio (AUC/MIC) of >17.9 (cSSI) and >6.96 (cIAI) describes the pharmacokinetic/pharmacodynamic (PK/PD) target of tigecycline [3,4]. The US Food and Drug Administration (FDA) does not recommend tigecycline as a first-line therapy of patients with severe infections. In a pooled analysis comparing tigecycline to other antibiotics in serious infections, there was an increased risk of death (4% (150/3788) vs. 3% (110/3646)), revealing an all-cause mortality of 0.6% (95% CI, 0.1% to 1.2%) probably due to progression of the infection [5]. This increased mortality was seen mostly in patients treated off-label for ventilator-associated pneumonia [5] and led to a black box warning by the US FDA (1 September 2010; 27 September 2013). However, with increasing resistance to first-line antibiotics and/or the lack of other treatment options, tigecycline is often one of a few last opportunities to treat severe infections. In addition to rational evaluation of the indication, the optimal dose is crucial to balance microbial eradication and tolerable side-effects. Several studies have reported increased microbiological eradication with higher tigecycline doses [6,7,8,9,10]. In addition, previously published studies have reported altered tigecycline pharmacokinetics in ICU patients [6,11]. Therefore, standard dosing may not be suitable for ICU patients, as compared to non-critically ill patients. Dose adjustment for drugs, which are metabolized or eliminated through the liver, is mostly performed by applying Child–Pugh Score (CPS) classification, as no single lab parameter can determine liver function and elimination capacity. The CPS originally assesses prognosis in chronic liver diseases and is generally classified in mild, moderate, and severe hepatic impairment, corresponding to scores of A (CPS_A_), B (CPS_B_), and C (CPS_C_). The tigecycline drug dossier informs about dosage in hepatic insufficiency, guiding no dose adjustment (100 mg initial dose, 50 mg q12h, i.v.) in mild or moderate impaired patients (CPS_A_, CPS_B_), but a maintenance dose reduction to 25 mg q12h for severe impaired patients with CPS_C_. Moreover, cirrhotic patients have a higher risk for infections with Gram-positive bacteria, which can also cause progression of liver failure. In addition to that, the severity of infections in these patients is often increased and correlated with a higher mortality [12]. Nevertheless, dosing decisions are often associated with uncertainty. On the other hand, bilirubin was previously related to tigecycline exposure, but has not been exploited for dose adjustments yet [13,14].

Hence, further data are required to elucidate the pharmacokinetics of tigecycline in liver-impaired critically ill patients. Therefore, we performed a population pharmacokinetic (popPK) analysis of tigecycline in this special patient population while evaluating liver-related clinical laboratory parameters (covariates) using a nonlinear mixed-effects modeling. Based on the developed popPK model, Monte Carlo simulations were used to investigate suitable covariates for dose adjustment and simulations of target attainment.

## 2. Results

### 2.1. Study Participants

This study recruited 39 patients, who contributed 283 timed plasma measurements of tigecycline to the pharmacokinetic (PK) model development. Table 1 summarizes patient characteristics, clinical laboratory data, infective pathogens, as well as underlying liver disease. Gram-positive bacteria or multiple pathogens mainly caused the infections. Two patients were undergoing renal replacement therapy (RRT), which was not considered as a relevant co-condition, as a previous study showed no relevant influence of RRT on tigecycline PK [13].

### 2.2. Pharmacometric Data Analysis

#### 2.2.1. Base Model

To analyze the plasma pharmacokinetics, nonlinear mixed-effects modeling was applied. A two-compartment model with linear disposition and elimination described tigecycline plasma pharmacokinetics and was superior to a one-compartment model to describe plasma pharmacokinetics (difference in Akaike Information Criterion (dAIC): −397). The residual unexplained variability was described by a proportional error model, and neither an additive error (drop of objective function value (dOFV): +206) nor a combined additive and proportional error model provided a better model fit (dOFV: −0.013). Interindividual variability (IIV) was supported on clearance (CL) (dOFV: −442), on central volume of distribution (V_c_) (dOFV: −48), and on peripheral volume of distribution (V_p_) (dOFV: −35). ETA-shrinkage was as low as 0% for CL and 13% for V_c_, indicating that most of the individuals contributed to these estimates of IIV. ETA-shrinkage was higher for V_p_ (43%), indicating that this IIV estimate was not supported by all subjects.

#### 2.2.2. Covariate Analysis

Exploratory graphical analysis and clinical relevance guided covariate selection for a stepwise covariate analysis procedure. For aspartate aminotransferase (AST), alanine aminotransferase (ALT), γ–glutamyltransferase (GGT), platelet count, and international normalized ratio (INR), no trends of individual PK parameters vs. these covariates were observed. Moreover, we did not include serum creatine into the covariate analysis, as estimated glomerular filtration rate (eGFR) carries more information about the kidney function. eGFR, total bilirubin (bilirubin_tot_), the maximum liver function capacity test (LiMAx test), the Model for End-Stage Liver Disease (MELD score), and CPS were tested on CL, and weight, sex, and age on V_c_ were considered as potential covariates in the population PK model. In the first step of the forward inclusion procedure, eGFR, bilirubin_tot_, CPS, as well as MELD score on CL and weight on V_c_ were significant covariates and reduced the IIV significantly (Table 2). From this starting point, three covariate models were built using either composite covariates (Models A and B) or continuous ‘raw’ covariates (Model C): Model A included CPS as a categorical covariate on CL to represent clinical practice. Model B included the MELD score, and Model C was built following the regular forward inclusion backward elimination procedure using the ‘raw’ covariates excluding the composite covariates Child–Pugh score and MELD score.

For Model A, the inclusion of the CPS as a categorical covariate was significant (dOFV: −11.7) and resulted in a descending order of tigecycline CL in relation to CPS. CPS_C_ patients showed a 50.1% reduced CL compared to patients with a score of CPS_A/B_. In addition, weight on V_c_ was significant (linear, dOFV: −9.9). IIV_CL_ was reduced from 48.2% to 41.8% and IIV_Vc_ from 85% to 70%. Supplementary Table S1 shows all final parameter estimates of that model.

For Model B, the MELD score, as a composite measure of liver and kidney function parameters, was a significant covariate on clearance (power, dOFV: −5.45). IIV_CL_ was reduced from 48.2% to 37.9%. IIV_Vc_ was reduced from 85% to 69.1% with weight on V_c_. Final model parameters are displayed in Supplementary Table S2.

For Model C, the final model included eGFR (linear, dOFV: −16.9) and bilirubin_tot_ (power, dOFV: −13.5) on CL and weight (linear, dOFV: −10.1) on V_c_. IIV_CL_ was reduced from 48.2% to 38.3% and IIV_Vc_ from 85% to 72.4%. Reduced eGFR and higher bilirubin_tot_ values corresponded to a lower CL to different extents. From the minimum to the maximum eGFR and bilirubin_tot_ value, the CL range totaled 5.62–10.5 L/h and 4.15–11.0 L/h, respectively. Supplementary Table S3 shows the final model parameter estimates of this model. Both the goodness-of-fit plots and prediction-corrected visual predictive checks (pc-vpc) indicated a good overall fit (Supplementary Figures S1 and S2). The median pc-vpc predictions and observations are best overlayed in Model A, and Model B showed a slight increase in confidence intervals at the 95th percentile.

The statistically best fitting model was Model C with an AIC of −952 and residual unexplained variability (RUV) of 12.4%, compared to Model A: −913 and Model B: −909. Nevertheless, the MELD score as a single covariate on clearance (Model B) could best reduce the IIV_CL_ (−10%) and, with that, to the same extent as Model A, which included two covariates on CL.

### 2.3. Monte Carlo Simulations

**Figure 1:**
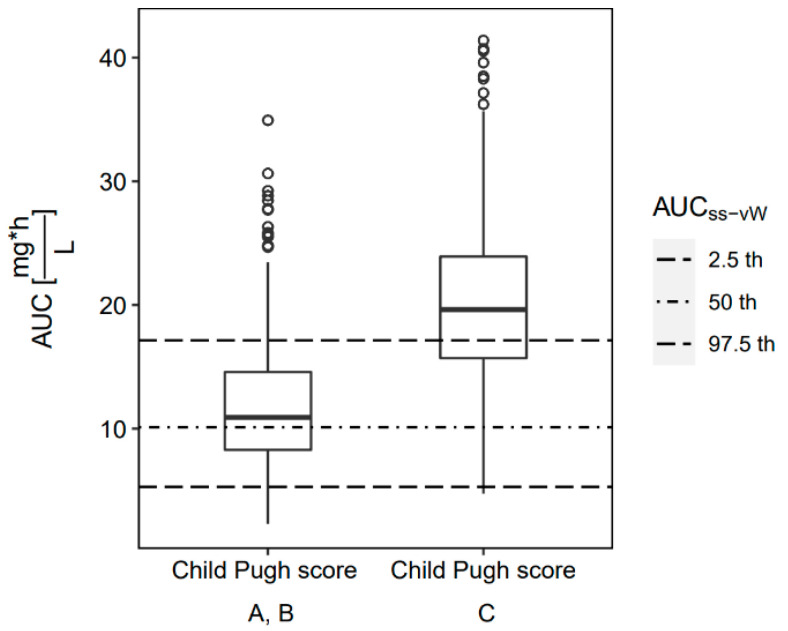
Simulated AUC_ss_ in patients with Child–Pugh A/B and C and standard-dose tigecycline (50 mg q12h MD) compared to the simulated AUC_ss-vW_ ‘reference’ of high-dose tigecycline (100 mg q12h MD) in non-critically ill patients.

Standard- (100 mg loading dose (LD), 50 mg q12h maintenance dose (MD)) and low-dose tigecycline (100 mg LD, 25 mg q12h MD) were simulated (n = 1000). Simulation results were used to explore dose adjustment strategies and probability of target attainment (PTA). In our study population, the simulated AUC_ss_ values after standard dosing using the best fitting covariate model (Model C) were 12.4 mg∙h/L in median (2.5th to 97.5th percentile: 4.10–27.1 mg∙h/L) and thus in the range of the median ‘reference’ AUC_ss_ values after high-dose tigecycline (100 mg q12h MD) in non-critically ill patients (10.1 mg∙h/L, 5.28–17.1 mg∙h/L) (AUC_ss-vW_ in Figure 1), yet more variable, indicating the need for dose adjustments. Indeed, if patients with CPS_C_ received the standard dose of 50 mg q12h, they displayed a 44.4% increased AUC_ss_ and a 117.5% increased steady-state C_min_ compared to CPS_A/B_. Therefore, only 33% of the patients would lie in the AUC_ss_ reference range due to overexposure (Figure 1).

**Figure 2:**
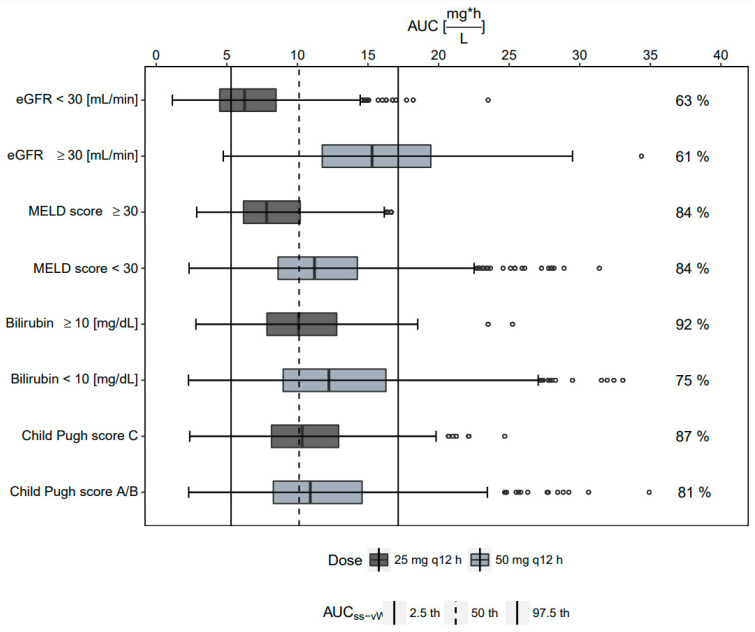
AUC_ss_ from dose-adjusted low-dose tigecycline (25 mg q12h MD) groups vs. non-adjusted groups receiving standard-dose tigecycline (50 mg q12h MD) in our cohort, compared to the 95% interval of 100 mg q12h MD tigecycline from van Wart et al. in non-ICU patients without hepatic impairment (AUC_ss-vW_, vertical lines). Optimal cutoffs for dose adjustment investigation were CPS_C_, total bilirubin ≥ 10 mg/dL, MELD score ≥ 30, and eGFR ≤ 30 mL/min. The quantity [%] of simulated individuals within the 95% interval of AUC_ss-vW_ is displayed.

Using CPS_C_ to guide a dose adjustment to a maintenance dose of 25 mg q12h MD provided the best alignment with the AUC_ss_ ’reference’ range, both in terms of the agreement of median AUC_ss_ and the fraction of simulated patients lying within the 2.5th and 97.5th AUC_ss_ interval (Figure 2). For the covariates MELD score, bilirubin, and eGFR, the optimal cut-offs for a dose reduction were found at ≥30, ≥10 mg/dL, or <30 mL/min, respectively.

However, the agreement of both median AUC_ss_ and the fraction of patients lying within the ‘reference’ range was considerably lower as compared to when using CPS_C_ to guide dose adjustment, and eGFR was found to perform worst (Figure 2).

### 2.4. Probability of Target Attainment

**Figure 3:**
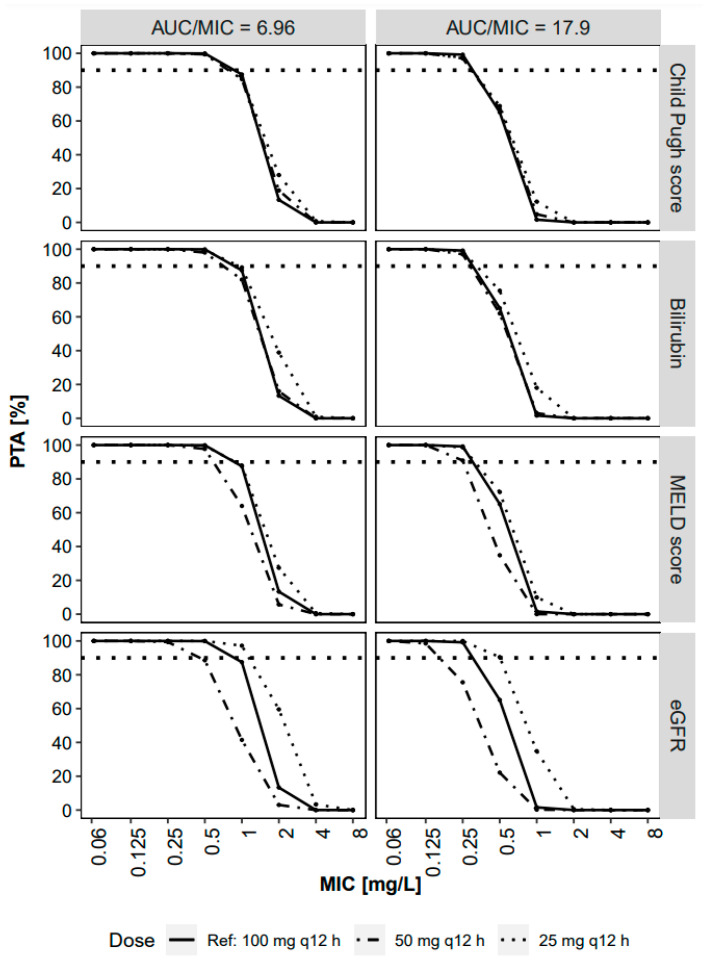
Probability of target attainment (PTA) analysis of AUC_ss_/MIC ratio ≥ 17.9 and ≥ 6.96 over minimal inhibitory concentration (MIC). Dose adjustment (25 mg q12h MD) was applied for individuals with bilirubin ≥ 10 mg/dL, MELD score ≥ 30, and eGFR ≤ 30 mL/min and compared to non-adjusted (50 mg q12h MD) groups, as well as Child–Pugh score-based dose adjustment. Horizontal dotted line denotes 90% PTA_90%_.

The alignment of the probability of PK/PD target attainment vs. MIC curve was highest when using the CPS-guided dosing. Nonetheless, all evaluated dose adjustment strategies provided a high (>90%) probability to attain the target for cIAI (PTA_90%_, AUC_ss_/MIC ≥ 6.96 [4]) for pathogens with a MIC ≤ 0.5. For the higher cSSI target (AUC_ss_/MIC ≥ 17.9 [3]), PTA_90%_ was attainted for pathogens with a MIC ≤ 0.25 (Figure 3).

### 2.5. Study Outcome

In this study, 8/39 patients (20%) showed clinical cure, 5/39 (13%) intermediate cure, and failure was observed for 26/39 (67%) patients. To evaluate clinical outcome, this analysis used odds ratios. The strongest correlation between therapy failure was observed for chronic liver disease (OR: 14.2 CI_95%_: 8.89–24.3), followed by serum creatinine (OR: 4.55, CI_95%_: 3.34–6.37), where the probability of therapy failure increased with increasing serum creatinine. eGFR, calculated by the CKD–EPI formula, was less significant (OR: 0.98, CI_95%_: 0.95–0.99), compared to solely serum creatinine. INR with an OR of 0.36 (CI_95%:_ 0.25–0.51), bilirubin_tot_ (OR: 1.30, CI_95%_: 1.22–1.39) and MELD score (OR: 1.13, CI_95%_: 1.10–1.16) were significant predictors of death. Neither the CPS_C_, AUC_24h_ of tigecycline, nor the pathogen causing the infection or other laboratory data were predictive for therapy failure or death.

## 3. Discussion

The present study aimed to assess the effects of different covariates on tigecycline exposure to evaluate their potential use as predictors for high and potentially supratherapeutic AUC values and their ability to guide dose adjustments in severe liver impairment. This patient cohort had highly variable AUC values, but the results agreed with previous findings, that CPS_C_ is able to guide a maintenance dose reduction from 50 mg to 25 mg q12h.

This study recruited a very vulnerable cohort with tigecycline treatment, which has not been represented well in the literature so far. Patients had different stages of acute and chronic liver impairment, exemplified by the strongly reduced typical CL of 7.52 L/h in our cohort compared to healthy volunteers, other ICU patients (e.g., 18.3 L/h [13], 22.1 L/h [15], 13.5 L/h [16]), and non-ICU patients (e.g., CL of 16.8 L/h [17] and 18.6 L/h [18]). Hence, simulated AUC_ss_ values were strongly increased at standard tigecycline dosing. Undoubtedly, safe and effective treatment is mandatory for these patients. However, dose adjustment for hepatically eliminated drugs is challenging, because the hepatic clearance cannot be solely determined by a single endogenous marker, such as creatinine clearance as a surrogate for renal drug clearance [19]. In our dataset, the CL reduction for patients with severe liver impairment with CPS_C_ was 50.1% and hence in line with the findings of Korth-Bradley et al. who found a CL reduction of 50.6% [20]. Hence, our results affirm the proposition by Korth-Bradley et al. to use CPS_C_ to guide dose adjustment. Furthermore, our covariate analysis identified bilirubin_tot_, eGFR, and MELD score as covariates of CL. Tigecycline is not exclusively metabolized, but substantially biliary excreted, explaining the correlation of bilirubin and MELD score to tigecycline clearance [21]. Moreover, the MELD score was recently suggested for dose adjustment in critically ill, liver decompensated patients, but was not directly compared to the CPS [22]. Furthermore, eGFR was a highly significant covariate in our study population. Kidney function is also affected with progression of liver disease [23], which could explain this strong correlation to tigecycline clearance. Korth-Bradley et al. observed a 20% decreased tigecycline clearance in renally impaired subjects, but no dose changes were recommended, as renal clearance accounts for only 20% of the total body clearance [24]. Our study results are in line with these findings, that strong eGFR reduction is not a signal to adjust the dose. Hence, CPS performed best to equalize tigecycline exposure in dose-adjusted (25 mg q12h MD) vs. non-adjusted patients (50 mg q12h MD), while maintaining an exposure equivalent to 100 mg q12h tigecycline observed in non-critically ill patients [18]. However, the CPS bears some limitations for use in clinical practice, as it is a composition of clinical variables and subjectively determined disease statuses [16,25]. In case CPS is unavailable, bilirubin_tot_ or MELD score might serve as alternatives to guide dose adjustment.

The dose adjustment algorithms evaluated in this study aimed to achieve an exposure profile equivalent to high-dose tigecycline (100 mg q12h MD) in non-ICU patients, as several studies have reported that the standard dose regimen of 50 mg q12h MD is not sufficient to achieve a reliable treatment success [6,10,26,27,28]. The current EUCAST MIC breakpoints for susceptible *Enterobacterales* and *Staphylococcus* is ≤0.5 mg/L (http://www.eucast.org/clinical_breakpoints/, accessed on 21 March 2022). Our simulations challenge this breakpoint as the PTA_90%_ results indicate sufficient target attainment at an MIC of 0.5 mg/L only for the cIAI target, but insufficient target attainment for the cSSI target even under high-dose tigecycline conditions. This is in line with the findings of Kispal et al., who concluded with escalating the dose to 150 mg i.v. q12h in patients with higher MICs [29].

The clinical data revealed MELD score and their components (bilirubin and SCR) to be predictive for survival in our collective. Contrarily, higher AUC_24h_ or AUC_72h_ values were not associated with a higher cure rate, indicating that other factors such as the underlying (liver) disease, organ dysfunction, and infection mostly affected the patient outcomes. According to that, previous studies of tigecycline use in critically ill patients associated the Sequential Organ Failure Assessment score (SOFA) with clinical failure [6,30]. Finally, further trials are warranted to enhance safety and efficacy of tigecycline treatment.

This study contributes significantly to the understanding of tigecycline pharmacokinetics in patients with different degrees of liver impairment. As a strength of this study, our study individuals showed a wide spread in covariate values: Well distributed covariate values are needed for derivation of reliable relationships of patient covariates with PK parameters. However, stepwise covariate modeling is known to have problems with selection bias and multiple testing [31], causing uncertainty in the covariate parameter estimates. Even if this study represents the largest in the liver-impaired patient collective with tigecycline monotherapy, a higher patient would be necessary to increase the accuracy of the estimates to further strengthen the conclusions. As another limitation of this study, the study documentation did not include assessment of encephalopathy, and hence, this variable was neglected in the CPS calculation. Several patients were mechanically ventilated, and therefore, assessment of encephalopathy is difficult in ICU patients with the presence of liver-unrelated comorbidity. On the other hand, the investigated dose adjustment with CPS was proven to be a robust covariate even with the related uncertainty.

Another limitation is that tigecycline itself can induce hepatotoxicity. However, the patients included in this study displayed pre-existing liver impairment before treatment with tigecycline was initiated. Moreover, the frequency of tigecycline-induced hepatotoxicity is low as transient elevations of serum aminotransferase levels occurs in only 2–5% of the patients [32], and tigecycline pharmacokinetics was stable over the therapeutic course. Hence, it is very unlikely that the observed high exposure of tigecycline in our collective is a consequence and not a cause of liver impairment.

## 4. Materials and Methods

### 4.1. Patients and Study Design

Patients from the surgical ICU of the Charité University Hospital, Berlin, Germany were recruited after ethical approval (EA4/022/13). Parts of the clinical raw data were already previously published [33], but neither were utilized for population PK modeling nor for the development of dose adjustment algorithms. The study cohort included adult patients older than 18 years with acute liver dysfunction secondary to sepsis, as well as patients with chronic liver dysfunction. Moreover, pathogens associated with the infection and clinical outcome were documented. Cure was defined as the resolution or significant improvement of signs and symptoms of the index infection, such that no additional antimicrobials or interventions were required. Clinical failure was defined as death due to infection prior to end of therapy, persisting or recurrent infection requiring additional intervention, or treatment with additional antimicrobials for ongoing symptoms of infection. Moreover, an intermediate cure was defined as trial data, which included death unrelated to the index infection, or extenuating circumstances that precluded classification as cure or failure.

In brief, patients received a loading dose of 100 mg administered as a 30 min infusion, followed by a maintenance dose of 50 mg q12h. Based on the treating physician’s assessment, eight patients received high-dose tigecycline with 100 mg q12h. Medical staff sampled at 0.3, 2, 5, 8, and 11.5 h after infusion at least 36 h after the start of therapy. Bioanalytical quantification of tigecycline plasma concentrations was performed, as previously described [34]. In addition to patient characteristics, clinical lab parameters included AST, ALT, GGT, SCR, eGFR according to CKD–EPI formula [35], albumin, bilirubin_tot_, platelet count, and INR. AST and ALT served for De-Ritis ratio calculation. This study also evaluated the MELD score, as well as the LiMAx test, which provides a direct measure of the metabolic capacity of the liver through phenotyping of CYP1A2 metabolism [36]. Furthermore, age, sex, body weight, and ascites status were documented. The CPS was calculated with the given parameters of bilirubin_tot_, albumin, INR, and ascites. Ascites were graded in none, mild (Grade 1), and moderate (Grade 2). The Child–Pugh Score calculations assumed no present encephalopathy, due to missing data. Moreover, no drug interaction of tigecycline was present and fluid balance was not considered. For evaluation of the clinical outcome, this analysis calculated odds-ratios using logistic regression for all laboratory liver parameters, the AUC_ss_ of tigecycline, and pathogens’ Gram type.

### 4.2. Pharmacometrics Analysis

#### 4.2.1. Base Model

This study used the nonlinear mixed-effects modeling program NONMEM^®^ (ICON, Gaithersburg, MD, USA, version 7.5), controlled by PsN 5.0 (Uppsala University, Sweden), for population pharmacokinetic analysis [37]. The population PK models were developed with first-order conditional estimation with interaction (FOCE+I). During model development, different compartments, and residual error models (additional, proportional, and combined) were tested. Inter-individual variability was assumed to be log-normally distributed and tested on all pharmacokinetic parameters, which were tigecycline CL, V_c_, inter-compartmental clearance (Q), and peripheral volume of distribution (V_p_).

#### 4.2.2. Covariate Analysis

An exploratory graphical analysis in combination with clinical relevance guided covariate selection. A stepwise method, based on the log-likelihood ratio test (forward inclusion: *p*-value < 0.05, backward elimination: *p*-value of <0.01), was applied. This study tested continuous covariate relationships such as power, linear, and exponential relationships on the respective pharmacokinetic parameters. In addition to the pure statistical criteria, we defined a strong IIV_CL_ reduction > 10% as a further specification of a covariate to be considered for evaluation of potential dose adjustment.

#### 4.2.3. Final Model Evaluation

We evaluated candidate models by graphical and numerical criteria (goodness-of-fit plots, prediction-corrected visual predictive checks, drop of objective function value, and difference in Akaike Information Criterion between two competing models (lower AIC indicates superior model)). Parameter uncertainty was evaluated using a log-likelihood profiling-based sampling-importance resampling routine (LLP-SIR), a technique for evaluating parameter uncertainty in small datasets [38].

#### 4.2.4. Simulations

Monte Carlo simulations (n = 1000) were utilized to simulate low- (100 mg LD, 25 mg q12h MD) and standard-dose (100 mg LD, 50 mg q12h MD) tigecycline. Covariates were resampled with replacement from the study participants to acknowledge potential correlations of the covariates in our study population. The candidate covariate models were exploited for dose adjustment. The target range for dose adjustment was defined by the AUC_24h_ range (10.12, 5.3–17.4 (50th, 2.5th–95th percentile). This range was achieved by simulating high-dose tigecycline (100 mg LD; 100 mg q12h MD) in non-critically ill patients using published pharmacokinetic information of the clinical study of van Wart et al. [18]. We chose to target the exposure after high-dose tigecycline, as it is associated with a more favorable clinical outcome than standard-dose [6,7,8,9].

Moreover, we performed a probability of target attainment analysis (PTA_90%_) using the target for cIAI (PTA_90%_, steady-state AUC_24h/MIC_ ≥ 6.96 [4]) and the higher cSSI target (steady-state AUC_24h_/MIC ≥ 17.9 [3]).

## 5. Conclusions

To summarize, dose reduction in severe liver impairment to a maintenance dose of 25 mg q12h was the best-guided CPS leading to AUC_ss_ values, which are equivalent to those found in non-ICU patients undergoing high-dose tigecycline (100 mg q12h MD), which was previously related to improve outcome. Bilirubin_tot_ and MELD score might serve as alternatives to guide dose adjustment but were inferior to CPS. Hence, a prospective evaluation of the tigecycline dosing strategy in patients with severe liver impairment is warranted.

## Figures and Tables

**Table 1:** Demographic and clinical patient characteristics. Clinical laboratory values are described by median with minimum and maximum values in square brackets.

Patient Characteristics	Total (n = 39)
Male (n)	13 (32.5%)
Female (n)	27 (67.5%)
Age (years)	62 [34, 85]
Weight (kg)	80.0 [44.5, 119]
**Clinical laboratory parameters**	
ALT (U/L)	33.5 [7.00, 928]
AST (U/L)	55.0 [13.0, 1300]
Total bilirubin (mg/dL)	2.64 [0.190, 18.6]
De-Ritis ratio	1.54 [0.167, 4.00]
ɣ-Glutamyltransferase (U/L)	120 [23.0, 1670]
INR	1.44 [0.970, 2.69]
LiMAx test [µg/h/kg]	170 [18.0, 596]
MELD score	18 [9.00, 37.0]
Serum creatinine [mg/dL]	1.09 [0.330, 3.31]
eGFR (CKD-EPI)	68.8 [17.2–149.8]
Thrombocytes (g/L)	148 [15.0, 777]
Child–Pugh score A (n)	21
Child–Pugh score B (n)	15
Child–Pugh score C (n)	3
**Underlying diseases**	
Acute liver impairment	22
Chronic liver disease	17
Klatskin tumor (type I, IIa, IIb, IV)	7
Liver abscess	3
Cholangiocarcinoma	2
Complicated cholecystitis	1
Liver cirrhosis	2
Hypoperfusion of the liver	1
Cholangiogenic sepsis	1
Ascites: none (n)	7
Ascites: Grade 1(n)	16
Ascites: Grade 2 (n)	16
**Microbiological isolates**	
*Enterococcus avium* (n)	1
*Enterococcus faecalis* (n)	4
*Enterococcus faecium* (n)	10
*Escherichia coli* (n)	4
*Klebsiella pneumoniae* (n)	1
MRSA (n)	2
*Staphylococcus epidermidis* (n)	6
VRE (n)	12

Abbreviations: ALT: Alanine aminotransferase, AST: Aspartate aminotransferase, LiMAx: Maximum liver function capacity, eGFR: estimated glomerular filtration rate, MELD: Model end-stage liver disease, INR: International normalized ratio, MRSA: Methicillin-resistant Staphylococcus aureus, VRE: Vancomycin-resistant Enterococci.

**Table 2:** Covariate analysis results from base model to first step in the forward inclusion, full models, and backward elimination.

	OFV	Implementation of Covariate Relationship	Model	dOFV	IIV	*p*-Value
Base model	−914.3	Two-compartment model with proportional error model			CL: 48.2% V_c_: 85%	
Forward inclusion	−928.6	linear	bilirubin_tot_/CL	−14.3	CL: 40.9%	<0.001
	−947.9	power	bilirubin_tot_/CL	−33.6	CL: 36.5%	<0.001
	−931.7	exponential	bilirubin_tot_/CL	−17.4	CL: 39.2%	<0.001
	−950.4	linear	eGFR/CL	−36.1	CL: 47.3%	<0.001
	−923.4	power	eGFR/CL	−9.0	CL: 43%	0.003
	−942.1	exponential	eGFR/CL	−28.5	CL: 48.4%	<0.001
	−930.8	linear	LiMAx test/CL	−16.5	CL: 59.1%	<0.001
	−926.5	power	LiMAx test/CL	−12.3	CL: 41.7%	<0.001
	−918.1	exponential	LiMAx test/CL	−3.81	CL: 54.6%	0.051
	−926.0	categorical	Child–Pugh/CL	−11.8	CL: 41.6%	<0.001
	−918.2	linear	MELD/CL	−3.94	CL: 39%	0.047
	−919.7	power	MELD/CL	−5.45	CL: 37.9%	0.019
	−918.1	exponential	MELD/CL	−3.83	CL: 38.7%	0.050
	−924.1	linear	WT/V_c_	−9.88	V_c_: 68.6%	0.002
	−920.9	power	WT/V_c_	−6.71	V_c_: 73.6%	0.009
	−917.9	exponential	WT/V_c_	−3.60	V_c_: 77.7%	0.058
	−921.4	linear	age/V_c_	−7.08	V_c_: 75.5%	0.008
	−916.9	power	age/V_c_	−2.60	V_c_: 85%	0.107
	−920.8	exponential	age/V_c_	−6.51	V_c_: 77.7%	0.011
	−918.1	categorical	sex/V_c_	−3.9	V_c_: 85.9%	0.048
Full model A	−936.0	Child–Pugh/CL (categorical) WT/V_c_ (linear)		−21.7	CL: 41.6% V_c_: 70.0%	
Full model B	−929.5	MELD/CL (power) WT/V_c_ (linear)		−15.3	CL: 37.9% V_c_: 69.1%	
Full model C	−974.4	eGFR (linear), bilirubin_tot_ (power), on CL WT (linear) on V_c_		−60.1	CL: 37.5% V_c_: 70.9%	
Backward elimination		linear	eGFR/CL	16.9		<0.001
		power	bilirubin_tot_/CL	13.5		<0.001
		linear	WT/V_c_	10.1		0.0014

Abbreviations: CL: Clearance, Vc: Central volume of distribution, IIV: Inter-individual variability, bilirubin_tot_: Total bilirubin, eGFR: Estimated glomerular filtration rate (CKD-EPI formula), LiMAx: Liver function capacity test, MELD: Model for end-stage liver disease, WT: Weight.

## Data Availability

Data are available on reasonable request due to restrictions.

## Supplementary Materials

The following supporting information can be downloaded at: https://www.mdpi.com/article/10.3390/antibiotics11040479/s1, Figure S1: Population or individual tigecycline predicted vs. observed concentration and conditionally weighted residuals or normalized prediction distribution errors (NPDE) vs. time; Figure S2: Visual predictive checks show prediction-corrected observations of tigecycline versus time after dose of the structural two-compartment base model and the covariate models (Model A–C); Table S1: Population pharmacokinetic model parameter estimates stratified by Child–Pugh score as a categorical covariate on clearance (Model A) and weight as a covariate on central volume of distribution V_c_; Table S2: Population pharmacokinetic model parameter estimates using the MELD score as a covariate on clearance as a power relationship and weight on V_c_ as a linear relationship (Model B); Table S3: Population pharmacokinetic parameter estimates of the final backward elimination model using raw covariate values (Model C).
