# Supplementary material for: Tigecycline Dosing Strategies in Critically Ill Liver-Impaired Patients
Source: Antibiotics (Basel). 2022 Apr 3;11(4):479. doi: 10.3390/antibiotics11040479 (PMC9028393; doi:10.3390/antibiotics11040479)
Supplement: Supplementary file 1 [file antibiotics-11-00479-s001.zip › antibiotics-1668178-supplementary.pdf]

Supplementary Materials

Figure S1 Population or individual tigecycline predicted vs. observed concentration (upper panel) and conditionally weighted residuals (CWRES) or normalized prediction distribution errors (NPDE) vs. time (lower panel); black line indicates the smoothed conditional mean incl. the 95th confidence interval (shaded area).

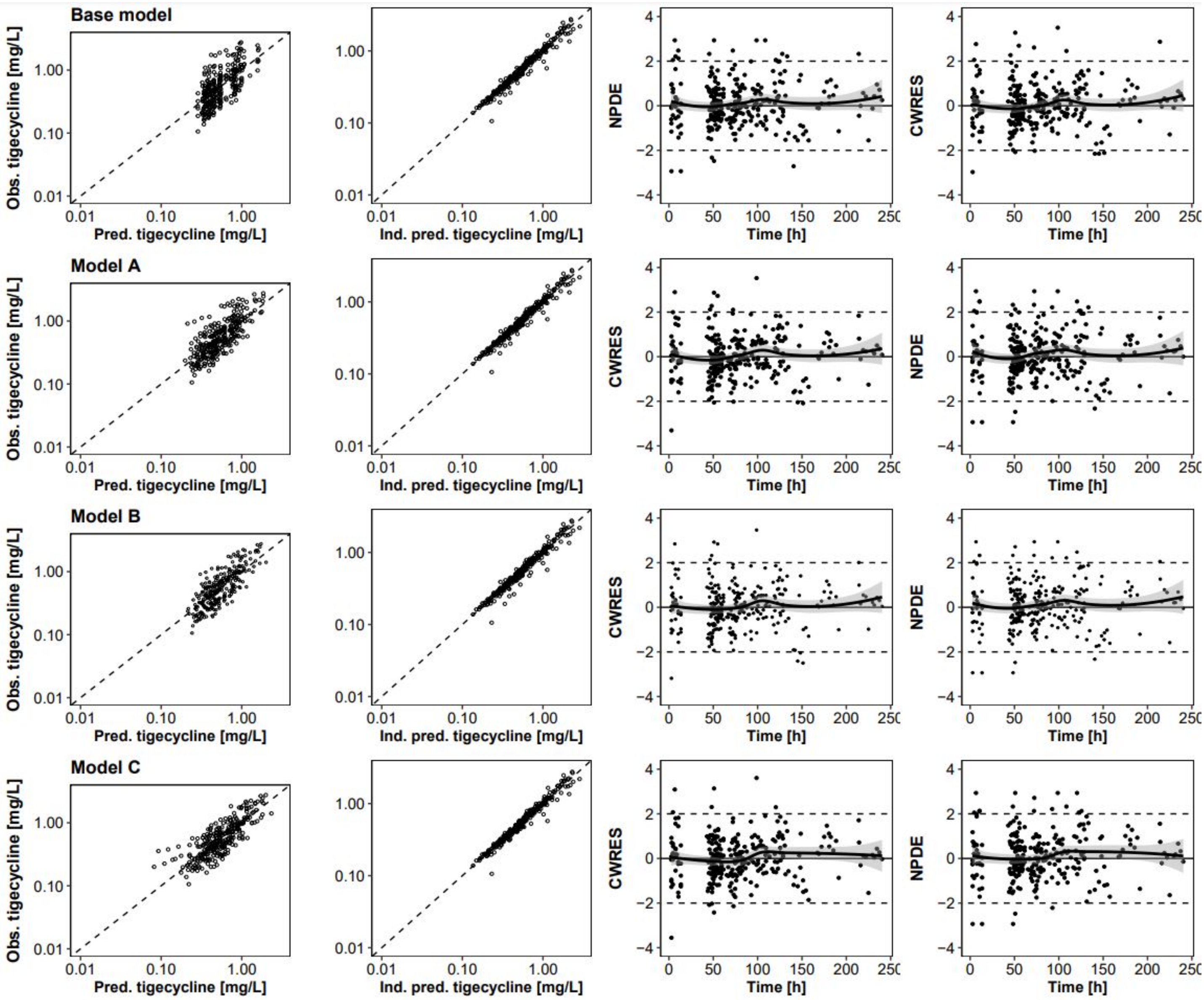

Figure S2 Visual predictive check show prediction corrected observations of tigecycline versus time after dose of the structural 2-compartment base model and the covariate models (Model A-C). All covariate models include beside the described covariate on clearance weight on central volume of distribution. The red lines show median, and 80% interval of the prediction corrected observations, black dashed lines describe the 80% interval of the simulated data’s percentiles. Blue shaded area shows the 95% confidence interval (CI) of the 5th and 95th prediction interval, red shaded area the 95%-CI of the median prediction.

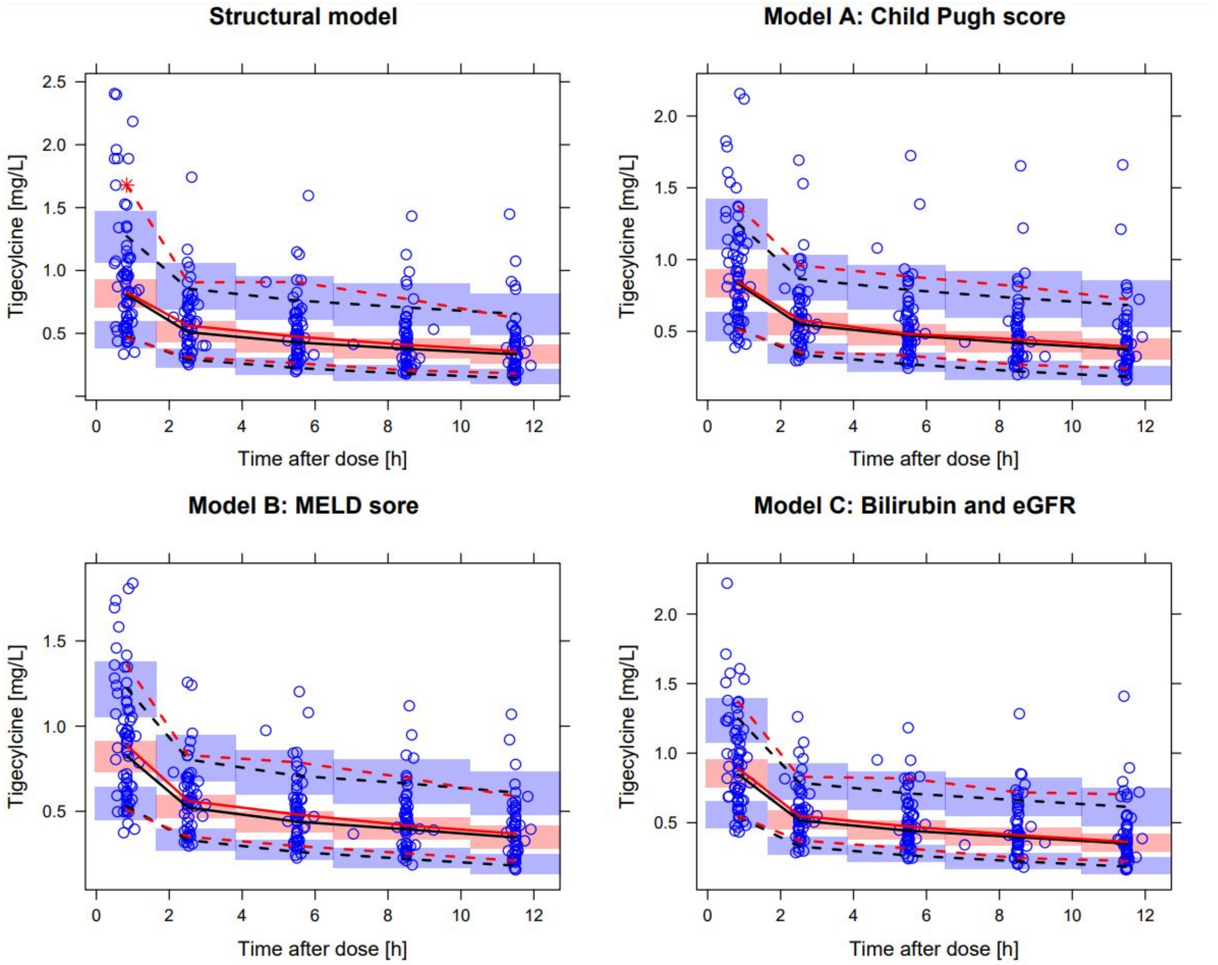

Table S1 Population pharmacokinetic model parameter estimates stratified by Child Pugh score as a categorical covariate on clearance (Model A) and weight as a covariate on central volume of distribution V_c (Eq. 1-2). Confidence intervals were determined by the log-likelihood profiling-based sampling importance resampling (llp-sir) method. Abbreviations: RUV: residual unexplained variability, TVVCL: Typical value of clearance. TVV_c: Typical value of central volume of distribution.

	**Explanation**	**Estimate**	**$CI_{95\%}$**	**RSE [%]**
$CL_{CPS\text{-}A}$ [L/h]	Clearance of individuals with Child Pugh score A	11.3	9.3 – 13.6	9.71
$CL_{CPS\text{-}B}$ [L/h]	Clearance of individuals with Child Pugh score B	7.69	6.61 – 9.16	8.46
$CL_{CPS\text{-}C}$ [L/h]	Clearance of individuals with Child Pugh score C	4.81	2.96 – 7.68	25.0
V_C [L]	Central volume of distribution	64.7	49.6 – 85.0	14.0
Q [L/h]	Intercompartmental clearance	48.4	42.1 – 56.3	7.46
V_P [L]	Peripheral volume of distribution	119	100 – 142	9.08
$\Theta_{Vc\text{ - }weight}$	Linear covariate parameter estimate of weight on V_c	$2.44 \cdot 10^{-2}$	$1.87 \cdot 10^{-2}$ – $2.77 \cdot 10^{-2}$	9.46
$IIV_{CHP\text{-}CL}$ [%]	Interindividual variability of Child Pugh A, B, C individuals on clearance	41.8	34.3 – 50.6	20.3
IIV_{Vc} [%]	Interindividual variability of central volume of distribution	70.0	52.6 – 85.5	24.6
IIV_{Vp} [%]	Interindividual variability of peripheral volume of distribution	30.5	19.7 – 40.6	34.5
RUV [% CV]	Residual proportional variability	13.5	12.2 – 15.0	5.21

IF(CPS = "A") TVCL = $CL_{CPS\text{-}A}$ Eq.(1)

IF(CPS = "B") TVCL = $CL_{CPS\text{-}B}$

IF(CPS = "C") TVCL = $CL_{CPS\text{-}C}$

$V_c = TVV_c \cdot (1 + \Theta_{Vc\text{ - }weight} \cdot (WT - 80))$ Eq.(2)

Table S2. Population pharmacokinetic model parameter estimates using the MELD-score as a covariate on clearance as a power relationship and weight on V_c as a linear relationship (Model B) (Eq. 3-4). Confidence intervals were determined by the log-likelihood profiling-based sampling importance resampling (llp-sir) method. Abbreviations: RUV: residual unexplained variability. TVVCL: Typical value of clearance. TVV_c: Typical value of central volume of distribution.

	**Explanation**	**Estimate**	**$CI_{95\%}$**	**RSE [%]**
CL [L/h]	Clearance	8.57	7.58 – 9.63	6.11
V_C [L]	Central volume of distribution	64.2	50.8 – 83.4	12.93
Q [L/h]	Intercompartmental clearance	48.7	42.2 – 56.0	7.23
V_P [L]	Peripheral volume of distribution	119	100 - 141	8.75
$\Theta_{CL\text{ - }MELD}$	Power relationship estimate of MELD-score on clearance	-0.453	$6.35 \cdot 10^{-1}$ – $2.78 \cdot 10^{-1}$	20.1
$\Theta_{Vc\text{ - }weight}$	Linear covariate parameter estimate of weight on V_c	0.024	0.019 – 0.028	9.42
IIV_{CL} [%]	Interindividual on clearance	37.9	30.4 – 48.3	25.1
IIV_{Vc} [%]	Interindividual variability of central volume of distribution	69.1	51.6 – 89.4	28.5
IIV_{Vp} [%]	Interindividual variability of peripheral volume of distribution	29.1	17.8 – 40.3	39.4
RUV [% CV]	Residual proportional variability	13.9	12.6 – 15.6	5.59

$$CL = TVCL \cdot (\text{MELD-score}/18)^{\Theta_{CL-MELD}} \qquad \text{Eq.(3)}$$

$$V_c = TVV_c \cdot (1 + \Theta_{Vc\text{ - }weight} \cdot (WT - 80)) \qquad \text{Eq.(4)}$$

Table S3 Population pharmacokinetic parameter estimates of the final backward elimination model using raw covariate values (Model C). Confidence intervals ($CI_{95\%}$) were determined by log-likelihood profiling-based sampling importance resampling (llp-sir); RSE: relative standard error. eGFR (CKD-EPI formula) and weight were included as a linear relationship bilirubin using a power function (Eq. 5-6).

Parameter	Explanation	Estimate	$CI_{95\%}$	RSE [%]
CL [L/h]	Total tigecycline clearance	7.52	6.68 – 8.46	6.03
V_c [L]	Central volume of distribution	63.4	49.8 – 83.0	13.4
Q [L/h]	Intercompartmental clearance	48.0	43.0 – 54.7	6.25
V_p [L]	Peripheral volume of distribution	120	102 – 144	8.84
IIV_{CL} [%]	Inter-individual variability of clearance	38.3	31.4 – 45.8	19.4
$IIV_{(Vc)}$ [%]	Inter-individual variability of central volume of distribution	72.4	54.0 – 90.7	25.8
IIV_{Vp} [%]	Inter-individual variability of peripheral volume of distribution	29.9	20.7 – 37.1	27.0
$\Theta_{CL-eGFR}$	eGFR on CL	$4.92 \cdot 10^{-3}$	$2.48 \cdot 10^{-3}$ – $7.83 \cdot 10^{-3}$	27.8
$\Theta_{CL-bilirubin}$	$Bilirubin_{tot}$ on CL	$2.12 \cdot 10^{-1}$	$3.09 \cdot 10^{-1}$ – $1.13 \cdot 10^{-1}$	23.7
Θ_{Vc-WT}	Weight on V_c	$2.45 \cdot 10^{-2}$	$2.0 \cdot 10^{-2}$ – $2.8 \cdot 10^{-2}$	8.43
RUV [%]	Residual unexplained proportional variability	12.4	11.3 – 13.8	5.21

$$CL = TVCL \cdot (1 + \Theta_{CL-eGFR} \cdot (eGFR - 68.8)) \cdot (bilirubin_{tot} / 2.64)^{\Theta_{CL-bilirubin}} \quad \text{Eq.(5)}$$

$$V_c = TVV_c \cdot (1 + \Theta_{Vc-weight} \cdot (WT - 80)) \quad \text{Eq.(6)}$$
